# Biomineralization Induced by Cells of *Sporosarcina pasteurii*: Mechanisms, Applications and Challenges

**DOI:** 10.3390/microorganisms9112396

**Published:** 2021-11-21

**Authors:** Yang Wu, Huimin Li, Yang Li

**Affiliations:** School of Chemical Engineering and Technology, Tianjin University, Tianjin 300072, China; 2014207404@tju.edu.cn (H.L.); ywu@tju.edu.cn (Y.L.)

**Keywords:** biomineralization, *Sporosarcina pasteurii*, urease, construction material, removal of heavy metals

## Abstract

Biomineralization has emerged as a novel and eco-friendly technology for artificial mineral formation utilizing the metabolism of organisms. Due to its highly efficient urea degradation ability, *Sporosarcina pasteurii* *(S. pasteurii)* is arguably the most widely investigated organism in ureolytic biomineralization studies, with wide potential application in construction and environmental protection. In emerging, large-scale commercial engineering applications, attention was also paid to practical challenges and issues. In this review, we summarize the features of *S. pasteurii* cells contributing to the biomineralization reaction, aiming to reveal the mechanism of artificial mineral formation catalyzed by bacterial cells. Progress in the application of this technology in construction and environmental protection is discussed separately. Furthermore, the urgent challenges and issues in large-scale application are also discussed, along with potential solutions. We aim to offer new ideas to researchers working on the mechanisms, applications and challenges of biomineralization.

## 1. Introduction

Biomineralization is a mineral-generating process catalyzed by the metabolism of organisms, which can harden the cells or the surrounding matrix to provide protection [1] and bracing [2]. Examples of biomineralization are widely found from microbes to animals, and can be witnessed throughout nature in the form of bones [3], shells [4], corals [5], as well as certain rocks and minerals [6]. More than 60 minerals are known to be formed by biomineralization, including ferric oxide, manganese oxide and other metal oxides.

According to the formation mechanism, biomineralization processes could be divided into two categories: biologically controlled mineralization (BCM) and biologically induced mineralization (BIM) [7]. In BCM, the location or structure of the synthetic biominerals is directly controlled by cells. As a typical example, magnetic iron biominerals can be precisely synthesized by magnetotactic bacteria as intracellular magnetosomes for sensing the geomagnetic field, and these particles have also been applied in the control and remediation of environmental pollution [8]. Arguably the most common biomineral, bone, is also synthesized in a precisely controlled manner by osteoblasts, which express alkaline phosphatase to hydrolyze pyrophosphate and provide inorganic phosphate for the formation of calcium phosphate in the bone matrix [9].

By contrast, BIM biominerals are formed due to chemical alterations of the local environment, which is induced by the metabolic activity of organisms. Thus, the locations and structures of BIM minerals are not controlled by cells. Recently, numerous BIM strategies have been developed using different organisms, including calcite precipitation by urea hydrolysis of ureolytic bacteria [10], carbonates formation by asparagine hydrolysis of *Bacillus megaterium* [11] and sulfide precipitation by sulfate reduction of sulfate-reducing bacteria [12,13]. Among these strategies, ureolytic bacteria are considered one of the most widely applicable due to their simple functional principle in urea hydrolysis catalyzed by urease and environmentally friendly performance in carbon neutrality [10,14]. Consequently, ureolytic biomineralization has been adopted for numerous applications in construction and environmental protection, such as self-healing concrete [15], bio-bricks [16], dust stabilization [17], ground improvement [18] and bioremediation [19].

**Figure 1:**
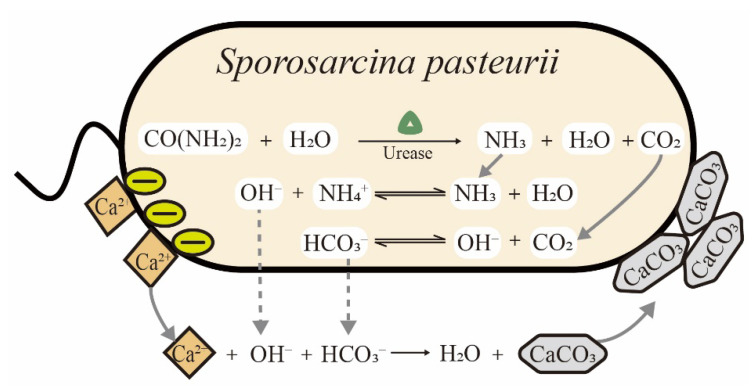
Schematic of microbially induced calcite precipitation (MICP) catalyzed by *S. pasteurii* cells.

*Sporosarcina pasteurii* (*S. pasteurii*) is a Gram-positive bacterium isolated from soil. It requires urea and ammonium for growth and has the ability to form spores in the harsh environmental conditions to enhance its survival. Among ureolytic microorganisms, *S. pasteurii* is the most frequently used strain in biomineralization due to its ultra-high urease activity. As shown in Table 1, the Km values of urease from different microorganisms are listed. The value of Km is the Michaelis constant, equal to the substrate concentration at which the reaction rate is half of the maximum rate in the enzymatic system. The Km is a measure of how efficiently an enzyme converts a substrate into a product. Notably, the urease of *S. pasteurii* is among the most active reported enzymes and constitutes up to 1% of a cell’s dry weight, leading to a distinct advantage for ureolytic biomineralization [20]. The process of microbially induced calcite precipitation (MICP) by *S. pasteurii* is schematically illustrated in Figure 1. Urease is expressed in cells and catalyzes the hydrolysis of urea to form ammonia and carbon dioxide. The ammonia leads to an increase in pH and carbon dioxide provides sufficient carbonate ions for calcium carbonate precipitation. Finally, the cell surface gathers calcium ions due to negatively charged groups and acts as the nucleation site of precipitates in MICP [21]. With the increasing consciousness of energy conservation and environmental protection in recent years, biomineralization using *S. pasteurii* cells has gained increasing attention among scientists, with potential applications in construction, environmental protection and medicine [22]. However, there are still challenges in both science and engineering that need to be addressed. For example, in studies on the dynamic growth of precipitates, conflicting mechanisms were still reported in recent years. Zhang et al. denied the role of the bacterial cell surface as nucleation sites during MICP [23], while Ghost et al. reconfirmed that precipitates are deposited on the cell surface using different methods [24]. In practical applications, many factors still need to be optimized for cost-effectiveness and scale-up. To our knowledge, the biomineralization induced by *S. pasteurii* cells have not been comprehensively summarized and reviewed in detail to date.

The purpose of this review is to give an overview of the biomineralization induced by *S. pasteurii* cells in terms of mechanisms, applications and challenges. We focus on the specific features of *S. pasteurii* cells contributing to biomineral formation, aiming to reveal the mechanism through which bacterial cells catalyze the biomineralization reactions. Furthermore, numerous applications in construction and environmental protection are summarized and discussed in detail, with the aim of expanding the potential to more possible fields. Finally, the urgent challenges and issues in large-scale application of a non-uniform biomineral structure, ammonia pollution and cost optimization are discussed, along with potential solutions. This review provides a reference to improve the scope and efficiency of studies on biomineralization.

## 2. Special Features of *S. pasteurii* Cells in Biomineralization

**Figure 2:**
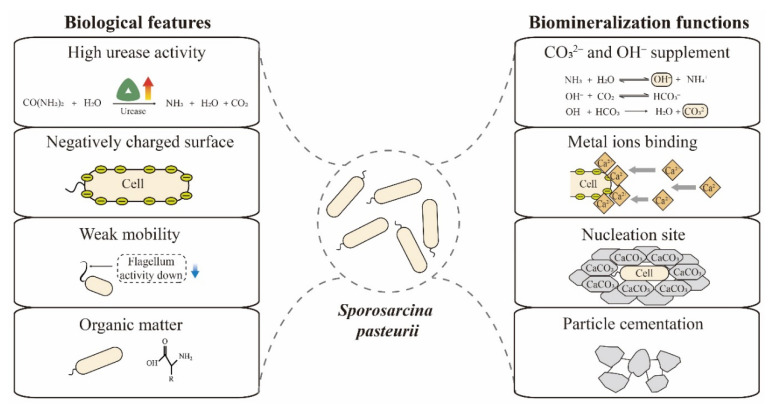
Relevant biological features and biomineralization functions of *S. pasteurii* cells.

Biomineralization efficiency is mainly determined by four factors—calcium, inorganic carbon, pH and nucleation sites [34]. *S. pasteurii* has multiple features that enable efficient biomineralization, including high urease activity, negatively charged surface, spore formation and weak mobility. As shown in Figure 2, these features play roles in the key dimensions of biomineralization. Here, we summarize and discuss these biological peculiarities and biomineralization functions of *S. pasteurii* cells.

### 2.1. Urea Hydrolysis

Since *S. pasteurii* was isolated from soil, its ultra-high urease activity has drawn extensive attention from researchers, and it can provide sufficient carbonate and hydroxyl ions for biomineralization (Figure 2). In order to investigate the mechanism of ultra-high urease activity, the structure, function and enzymatic properties of urease were researched comprehensively [35]. The urease from *S. pasteurii* cell is a nickel-dependent enzyme with a trimeric structure, in which each monomer contains one large (α subunit UreA) and two small subunits (β subunit UreB and γ subunit UreC) [36]. Moreover, four auxiliary proteins, UreD, UreE, UreF and UreG, are also necessary for urease assembly. The assembly process consists of three steps according to the reported hypothesis [20,37]. Firstly, the three-subunit complex, UreABC, is expressed from the genome and self-assembled. At the same time, the auxiliary protein complex, UreDFG, is also expressed and formed as another three-subunit complex. Subsequently, UreABC–UreaDFG is assembled as the final supercomplex. Finally, the metallochaperone UreE transfers the nickel ion into the active center of the UreABC–UreDFG complex and completes the activation of ureolytic activity [38].

Among the auxiliary proteins, UreE is the only nickel-binding protein and the key factor for urease activation. However, the definite dynamics of nickel loading and urease assembly has not been fully understood to date. In computational simulation studies, Carlsson et al. presented a DFT-based calculation method to study the urease mechanism and found that the binding site of the urea substrate included the nickel ion in the active center [39]. In experimental studies, Won et al. reported that the conserved side chain at the C-terminus of UreE serves as a ligand of the nickel ion. Furthermore, the nickel ion content of urease decreased dramatically in the absence of UreE, leading to enzyme inactivation [40]. Similarly, Scott et al. performed knockout studies of partial sequences in the *ureE* gene, causing about a 50% decline in ureolytic activity [41]. By contrast, there are limited reports investigating the other auxiliary proteins, including UreD, UreF and UreG. Liu et al. expressed the UreABC genes in *E. coli* and improved urease activity about 5–6 times by the co-expression of UreDFG, demonstrating the importance of UreDFG for urease activity [42].

**Figure 3:**
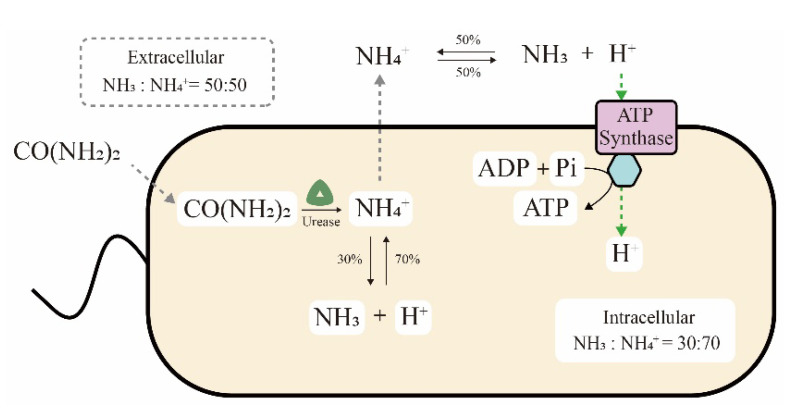
ATP generation through the proton electrochemical potential induced by urea hydrolysis.

The ultra-high ureolytic activity is also related to the metabolic mechanism of *S. pasteurii* cells. On the one hand, generation of the proton electrochemical potential by ureolysis has been speculated to be an ATP production pathway in *S. pasteurii* cells, as shown in Figure 3 [43,44]. In detail, NH_4_^+^ produced from intracellular urea hydrolysis spreads to the extracellular space. The reversible balance ratios of NH_4_^+^ generating NH_3_ and H^+^ are different in intracellular and extracellular environments, at 70:30 and 50:50 of NH_4_^+^ vs. NH_3_, respectively. Finally, the proton concentration gradient of more extracellular H^+^ results in a proton motive force to drive ATP synthase for energy generation. Recently, this hypothesis was further confirmed by transcriptome analysis. Ma et al. reported that the genes for ATPase synthesis were upregulated in the MICP process or a urea-deficient environment, implying the relationship between ATP and urease [21]. Pei et al. also elucidated that urea hydrolysis might promote the synthesis of intracellular ATP and the cells could not grow normally in the absence of urea [45]. On the other hand, a survival advantage was also observed in *S. pasteurii* cells that utilize the urea hydrolysis process. Specifically, massive amounts of NH_3_ and OH^−^ were generated during urea hydrolysis, increasing the ammonia concentration and pH value in the environment. Consequently, competing organisms that are sensitive to ammonia or an alkaline solution would be killed, while *S. pasteurii* cells could survive due to their high alkali and ammonia resistance [46].

Recently, strains with enhanced urease activity were obtained by plasma mutagenesis and UV irradiation, significantly increasing the amount of precipitated calcium carbonate in MICP [47,48]. Overall, although the urease dynamics and metabolic mechanism of *S. pasteurii* cells still need to be elucidated more clearly, the ultra-high ureolytic activity has been investigated and utilized for carbonate and hydroxyl ion supply in biomineralization.

### 2.2. Precipitate Nucleation

Nucleation is a key process in biomineral formation, which requires nucleation sites and sufficient reactive ions to form the insoluble precipitate [49]. Coincidentally, *S. pasteurii* cells could provide these conditions efficiently. In biomineralization, the negatively charged surface of bacterial cells could bind cations (metal ions) and create a regional ion-saturated microenvironment to satisfy the nucleation requirements [50]. Moreover, *S. pasteurii* cells have been reported to have more negative surface charges than non-mineralizing bacteria such as *Bacillus subtilis*, *Staphylococcus aureus* and *Escherichia coli*, which could greatly benefit the nucleation step [21].

The bacterial cell surface has been revealed to act as a nucleating site in biomineralization through various approaches. Utilizing ultramicrosensor detection, Harris et al. observed the precipitation of biominerals on the surface of *S. pasteurii* cells during the MICP process, suggesting the involvement of the cell surface in the nucleation process [51]. Transcriptome analysis revealed decreased flagellar gene expressions in *S. pasteurii* cells during biomineralization, which could inhibit cell mobility and improve the effectiveness of cells serving as the nucleation sites [21]. Moreover, Ghost et al. found conclusive evidence that nanoscale crystals formed on the bacterial cell surface using scanning electron microscopy (SEM) and transmission electron microscopy (TEM) [24]. Nonetheless, some contradictory perspectives were still reported in recent years. Zhang et al. denied the role of the bacterial cell surface as a nucleation site during MICP based on an in situ real-time study at the single-cell resolution with the recorded grain sizes, which was contradictory to the above investigations [23]. Hence, the nucleation process induced by bacterial cells may be more complicated than reported, requiring more definite studies in the future. For example, the quantitative parameters of nucleation were required in quantitative analysis. However, to the best of our knowledge, microscopy techniques, SEM and TEM, were the main methods in biomineralization researches. Thus, the recorded grain sizes were the only quantitative parameters in this process. Indeed, more quantitative parameters need to be developed in the future.

### 2.3. Biomineral Cementation and Spore Formation

Microbial cementation is a fundamental step in the binding of loose particles in the form of sandstone in some biomineralization applications, which can improve the mechanical properties of biominerals. However, such cementation was not witnessed in traditional chemical precipitation, suggesting additional effects of the microbial cells in biomineral cementation [52].

In order to investigate the cementation mechanism of microbes in biomineralization, a serious of efforts have been made in recent years. Rong et al. compared different sand columns prepared using biological or chemical approaches and found that only the biological sand column transformed into a solid material with remarkable strength [53]. Subsequently, mechanisms of biomineral cementation were explored by numerous approaches, including nuclear magnetic resonance (NMR), X-ray photoelectron spectroscopy (XPS), infrared spectroscopy (IR) and TEM. The results indicated that additional hydrogen bonds were generated between particles during the biomineralization process, which might contribute to the cementation function [54]. Based on a study of the cementation interface of bio-cement, Qian et al. concluded that hydrogen bonds were formed between the hydroxide radicals of polypeptides on the cell surface and oxygen atoms of oxide minerals, so that the cementation was a result of the combined effects of microbes and minerals. For example, in the biomineralization of a sand column, the hydrogen bonds were supposed to be formed between the oxygen in the silica and the peptides in the organic matter for biomineral cementation [55].

Additionally, spore formation of the Gram-positive *S. pasteurii* cells also improves their tolerance of extreme environmental conditions, such as nutrient deficiencies, extreme temperatures and pH values [56]. This ability to form spores is indirectly related to biomineralization. However, such long-term viability of bacteria opens the possibility of long-term biomineralization reactions in extreme applications, including self-healing concrete [57] and the bioremediation of metal ions [58]. Notably, the non-spore-forming Gram-positive bacterium *Staphylococcus pasteurii* was also isolated and tested in biomineralization investigations. Compared with *S. pasteurii* cells, the survival time of *Staphylococcus pasteurii* in the soil was much shorter due to the absence of spore formation [59].

## 3. Applications of Biomineralization

With the discussed advantages of *S. pasteurii* cells, ureolytic biomineralization has been successfully applied in construction and environment protection, improving the scope and efficiency of environmentally friendly techniques. Here, we discuss the challenges and opportunities of these current and emerging applications.

### 3.1. Construction Applications

Undeniably, most applications of biomineralization technology can be found in construction. Extensive studies have been carried out for potential applications in both the construction and repair of buildings using *S. pasteurii* cells, including the typical examples of self-healing concrete and bio-bricks.

**Figure 4:**
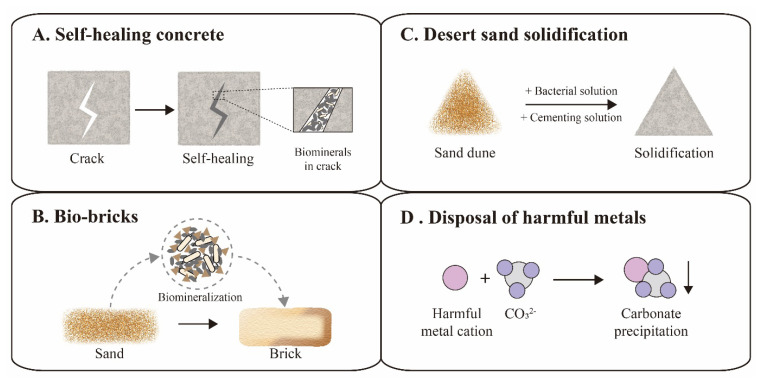
Applications of ureolytic biomineralization. Self-healing concrete (**A**) and bio-bricks (**B**) are representative cases in the construction field. Desert sand solidification (**C**) and disposal of harmful metals (**D**) are representative cases in the environmental protection field.

The increasing cracks and holes in aging concrete structures can seriously compromise the safety and tightness of constructions, leading to expensive repairs or reconstruction. In order to address this issue, traditional concrete was suggested to be replaced with a novel self-healing concrete, which could automatically heal the cracks through biomineralization (Figure 4A) [60,61]. The first examples of self-healing concrete were composed of *S. pasteurii* cells, calcium ions, nutrients, concrete and other supplements. The bacterial spores could survive up to 10 years in the concrete matrix [61]. Once cracks appear, the rainwater that seeps into the cracks would activate spores to fill the space of the cracks via a biomineralization reaction, achieving the goal of self-healing [62]. However, certain additives in self-healing concrete, such as bacterial cells, nutrients and calcium supplements, might also compromise the performance of the concrete [63]. Recently, several studies focused on the optimization of self-healing concrete for performance enhancement. For instance, Chen et al. investigated the influence of the *S. pasteurii* cell concentration on the self-healing properties, and suggested 2 × 10^6^ cells/mL as the proper bacterial concentration [64]. Kang et al. proposed calcium lactate as nutrient and calcium supplement for *S. pasteurii* cells, avoiding damaging the hardness of the concrete due to traditional saccharide nutrients [65]. Furthermore, some additional additives were included to improve self-healing concrete, including polyurethane, silica gel and air-entraining agents [66,67,68,69].

In addition to self-healing concrete, brick manufacturing has also applied novel biomineralization technology, called bio-bricks. In 2012, the BioMason company was founded for bio-brick research and development using *S. pasteurii* cells [70]. Their patented bio-brick “bioLITH ^®^ tiles” are available now in practical building construction. In the manufacturing process, these bio-bricks are cultured in custom shaped molds, where sand is mixed with bacterial cells and cemented into a whole by the biomineralization reaction (Figure 4B) [71]. Recently, the bio-brick technique was developed for better performance and cost-effectiveness by numerous efforts. Li et al. investigated the effects of different additives on the properties of bio-bricks, and found that fiber supplements could increase the compression strength of the bio-brick by 50–70% [72]. Lambert et al. successfully grew the world’s first bio-brick from human urine instead of chemical urea, reducing production costs [73].

Additionally, the technology of biomineralization has been applied in oil-well plugging [74], solidification of riverbanks [75] and rescue of historical buildings [76]. These aging structures have undergone deterioration due to chemical and physical weathering, requiring restoration of cracks and holes. Then, *S. pasteurii* cells and supplements were injected and utilized to generate biominerals as a stuffing or adhesive for the damaged areas, accomplishing the purpose of a life-expectancy extension of existing structures.

### 3.2. Environmental Applications

In addition to applications in the construction industry, biomineralization was also employed in environmental protection applications, such as desert solidification and removal of harmful metals. Desert expansion and resulting loss of farmland is a worldwide problem, requiring widely applicable solutions [77]. Although numerous chemical approaches have been proposed for desert sand stabilization, the costs and labor intensity still need to be reduced in view of the vastness of desert areas. Recently, biomineralization was developed as an environmentally friendly and cost-effective method for sand stabilization by simulating natural processes (Figure 4C). Katebi et al. spread a cementation solution containing *S. pasteurii* cells on the sand surface, which resulted in a stable sand crust at the field scale in the desert [78]. A similar strategy was also applied for the solidification of fly ash into a green sustainable material, increasing the compressive strength up to 43.75% [79]. Looking forward, Larsson et al. envisioned bold plans to solidify Sahara sand to build comfortable shelters for humans and prevent the spreading of the desert, illustrating the vast potential of biomineralization in environmental stewardship [80].

Harmful metal disposal is emerging to be a great challenge to environmental remediation, which seriously threaten human health [81]. Fortunately, a biomineralization reaction induced by *S. pasteurii* cells could provide sufficient CO_3_^2−^ ions to integrate these metal cations into undissolving carbonate for easy removal (Figure 4D) [82]. Soils contaminated with strontium (Sr) or lead (Pb) were successfully removed by biomineralization of *S. pasteurii* cells with more than 99% efficiency [83]. Recently, multiple heavy metals, such as cadmium (Cd), zinc (Zn) and copper (Cu), were also reported to be eliminated from soils using biomineralization technology [84,85]. This bioremediation technology has become an attractive solution for the removal of harmful metals. In general, biomineralization mimics the natural biogeochemical carbon cycle, which could revolutionize sustainable industries and create an environmentally friendly society in the future.

## 4. Challenges and Issues

Although there are increasing numbers of reports demonstrating the potential of biomineralization applications in numerous fields, large-scale commercial projects are still restricted by several key limitations, including a non-uniform biomineral structure, ammonia pollution and cost optimization. In this part, the challenges and issues of biomineralization in practical applications will be summarized and discussed.

### 4.1. Non-Uniform Biomineral Structure

Uniformity is a basic requirement of products with stable performance in a wide range of applications. However, in large-scale biomineralization, different degrees of a reaction always appear in different areas of the product, leading to non-uniform structures with uncertain performance [86]. Qian et al. investigated large-scale biomineralization by injecting *S. pasteurii* cells and supplementary solutions into a 125-L sand column. Analysis of both the calcite content and compressive strength of the sand column indicated the uneven distribution of biominerals, whereby there was reduced biomineralization farther from the injection point [87]. At the injection point, biominerals were quickly formed with freshly injected cells and solutions, blocking fluent inflow of subsequent solutions and leading to non-uniform biomineralization reactions [88].

To address this intractable issue, several strategies were proposed recently. On the one hand, the concentrations of ingredients in the injection solutions were optimized to reduce the reaction rate for uniform biomineralization. Salwa et al. reduced the Ca^2+^ concentration and obtained a one-meter homogeneous sand column with 5.9 MPa of compressive strength [89]. Similarly, a bacterial solution with an OD_600_ of 1.0 was selected to prepare a more uniform biomineral with higher strength [90]. On the other hand, efforts were also made in the optimization of the injection of bacterial or supplementary solutions [91]. Rong et al. confirmed that a slow rate of bacterial solution flow (5 mL/min) contributed to uniform product mineralization by preventing the blockage caused by a quick reaction at the injection point [92]. Tobler et al. presented a new method of injecting bacterial and supplementary solutions at different positions of the sand column, which resulted in a uniform distribution of biominerals in the product [93]. However, practical field-scale verification of these laboratorial strategies is still needed before commercial applications can be considered [94].

### 4.2. Ammonia Pollution

Biomineralization via urea hydrolysis induced by *S. pasteurii* cells produces large amounts of ammonia as a by-product, which leads to environmental pollution with toxic effects on ecosystems, vegetation and human health [95]. To overcome this problem, researchers also tested non-ureolytic bacteria. For example, Zhu et al. replaced *S. pasteurii* with the photosynthetic bacterium *Synechococcus* PCC8806, to generate HCO_3_^−^ for carbonate precipitation without ammonia emissions [96]. Notably, a ureolytic biomineralization method without ammonia emission was recently proposed based on struvite precipitation induced by *S. pasteurii*, which locks ammonium in the final product, avoiding pollution [97]. This might be an appropriate solution to encourage large-scale applications of biomineralization after the necessary validation studies.

### 4.3. Cost Optimization

On the laboratory scale, high-grade reagents such as nutrient sources and pure urea were used in most studies of biomineralization, irrespective of costs. However, the cost of commercial projects is a crucial limitation in the large-scale application of biomineralization, requiring economical alternatives to these laboratory reagents. Fortunately, several cost-effective alternatives were discovered and analysed. As a nutrient source, some waste products of the food industry are suitable substitutes typically found at a bargain price and in large amounts, including whey powder from cheese production [98,99], lactose from the dairy industry [100] and corn steep liquor from corn starch processing [101]. Additionally, chicken manure was also utilized as a cultivation medium for *S. pasteurii* cells [102]. Kitchen waste is also an alternative nutrient source for the culture of bacterial cells [103]. As a replacement for highly pure synthetic urea, both pig urine and human urine were investigated in biomineralization for cost reduction [16,104]. Even distilled water was successfully replaced with Caspian seawater, saving costs during the large-scale distilled water preparation of biomineralization [98]. Looking forward, more sources and proportions of other components, such as calcium and supplementary reagents, still require further optimization for low-cost and high-quality applications [90,105].

## 5. Conclusions

Biomineralization induced by *S. pasteurii* cells has emerged as a powerful technology with booming research on its mechanisms, applications and challenges, which were firstly summarized in this review. Due to the advantages of ultra-high urease activity, negatively charged surface, spore formation and weak mobility, *S. pasteurii* cells are considered an ideal strain for the key processes of urea hydrolysis, precipitating nucleation and biomineral cementation in ureolytic biomineralization. Moreover, numerous novel applications, such as self-healing concrete, bio-bricks, desert sand solidification and disposal of harmful metals, were developed rapidly in the construction and environmental protection fields, illustrating the vast future potential of biomineralization. In large-scale engineering, the challenges and issues of a nonuniform biomineral structure, ammonia pollution and cost optimization were discussed in terms of current progress and urgently needed further solutions. Efforts should be made to shore up these weak spots in future studies. By emphasizing these crucial aspects of biomineralization induced by *S. pasteurii* cells, this review is intended to provide a bridge between current research and future commercial-scale applications in this rapidly developing field, assisting both scientists and engineers in their studies on mechanisms, applications and challenges.

## Figures and Tables

**Table 1:** Urease activity of different microorganisms.

Microorganism	Km of Urease (mol/L)
*Sporosarcina pasteurii*	40–130 [25]
*Brevibacterium ammoniagenes*	18–72 [26]
*Providencia stuartii*	10.50–71 [27]
*Proteus mirabilis*	13 [28]
*Arthrobacter oxydans*	12.50 [29]
*Staphylococcus saprophyticus*	7.36 [30]
*Klebsiella aerogenes*	2.80 [31]
*Aspergillus nidulans*	1.33 [32]
*Spirulina maxima*	0.12 [33]
